# Interpretable and explainable predictive machine learning models for gastric retention before gastroscopy

**DOI:** 10.3389/fmed.2026.1898316

**Published:** 2026-08-27

**Authors:** Yun Dai, Huan-Zhong Su, Hong-Bin Zhang, Jin-Hai Chen, Guo-Xing Xu, Hai-Xing Wang

**Affiliations:** 1 Department of Endoscopy, The First Affiliated Hospital of Xiamen University, School of Medicine, Xiamen University, Xiamen, China; 2 Department of Ultrasound, The First Affiliated Hospital of Xiamen University, School of Medicine, Xiamen University, Xiamen, China

**Keywords:** gastric retention, gastroscopy, machine learning, predictive model, risk stratification

## Abstract

**Background:**

Early detection of gastric retention (GR) is challenging in clinical practice. We aimed to develop and validate interpretable machine learning (ML) models for predicting GR risk using routinely available clinical features.

**Methods:**

This retrospective study enrolled 3,233 consecutive patients (training cohort: *n* = 2,263; validation cohort: *n* = 970) who underwent gastroscopy between January 2022 and December 2024. To address class imbalance, the Synthetic Minority Over-sampling Technique (SMOTE) was applied prior to model development. Optimal features were selected via least absolute shrinkage and selection operator (LASSO) regression within the SMOTE-enhanced training cohort, with different predictive models constructed using nine machine learning (ML) algorithms. Ten-fold cross-validation on SMOTE-enhanced training cohort was used for hyperparameter tuning. Model performance was primarily assessed on the validation cohort using the area under the receiver operating characteristic curve (AUC), calibration plots, and decision curve analysis (DCA).

**Results:**

LASSO regression identified 12 clinically actionable predictors from 19 candidate variables, which were subsequently used to develop nine ML models. Among these models, the extreme gradient boosting (XGBoost) model achieved the highest AUC value of 0.710 (95% CI: 0.681–0.738) in the validation cohort. Decision curve analysis revealed that the XGBoost model exhibited superior clinical utility compared to other models. Shapley Additive Explanations (SHAP) identified the use of anticholinergic drugs, pyloric obstruction, analgesic use, systemic diseases, and a history of gastrointestinal surgery as the top five important predictive features.

**Conclusions:**

This study establishes an interpretable GR risk stratification model using routinely available preoperative variables. The model may serve as an auxiliary screening aid for pre-gastroscopy risk assessment.

## Introduction

Gastric retention (GR) is a clinically significant condition characterized by delayed food emptying and diagnosed during gastroscopy (1). Although fasting for 6–8 h is routine before gastroscopy, it does not seem to be effective for some patients. Previous studies have shown that patients with diabetes and a history of gastrointestinal surgery are prone to GR (2–5). A systematic study indicated that GR can be detected in approximately 40% of patients with type 1 diabetes and 20% of patients with type 2 diabetes (6). Patients with GR face a high risk of aspiration, which can lead to pneumonia or even asphyxiation, especially when undergoing anesthetized gastroscopy. Moreover, traditional risk stratification methods lack precision in identifying high-risk individuals, particularly in populations with heterogeneous metabolic profiles or polypharmacy use. With the global increase in diabetes, obesity, and surgical interventions, there is an urgent need for predictive tools that integrate multifactorial clinical data to enable early risk stratification and personalized management.

Recent advances in machine learning (ML) have demonstrated transformative potential in clinical prediction tasks. For instance, interpretable ML models have successfully predicted conditions such as extended-spectrum *β*-lactamase (ESBL)-positive urinary tract infections and tuberculosis development by leveraging routine clinical variables, achieving high accuracy (AUC >0.90) and clinical interpretability (7, 8). Notably, ensemble algorithms like XGBoost have emerged as robust frameworks for handling imbalanced datasets and capturing nonlinear interactions among predictors, as evidenced by their superior performance in predicting cardiovascular drug use and metabolic risk trajectories (9, 10). A key challenge in developing ML models for GR lies in balancing predictive performance with clinical interpretability. Recent studies highlight the value of explainable AI techniques, such as Shapley Additive Explanations (SHAP), in elucidating feature contributions and guiding actionable interventions (11, 12). For example, SHAP analysis in predicting Parkinson's disease and surgery-related cardiovascular events revealed modifiable risk factors, such as medication use and biomarker thresholds, which informed targeted therapeutic strategies (13–15).

However, the application of ML to GR prediction remains underexplored. Aspiration caused by gastric retention can lead to severe pneumonia or even asphyxiation, which exacerbates the disease burden on patients and the social medical burden resulting from repeated examinations. Herein, we retrospectively analyzed a large number of patients with GR to develop and validate an interpretable ML model for GR risk prediction using routinely collected clinical data. Information obtained from this large-scale study aligns with emerging trends in precision medicine, where ML-powered tools are increasingly deployed to optimize diagnostic workflows and reduce complications in high-risk populations.

## Materials and methods

### Patients

**Figure 1:**
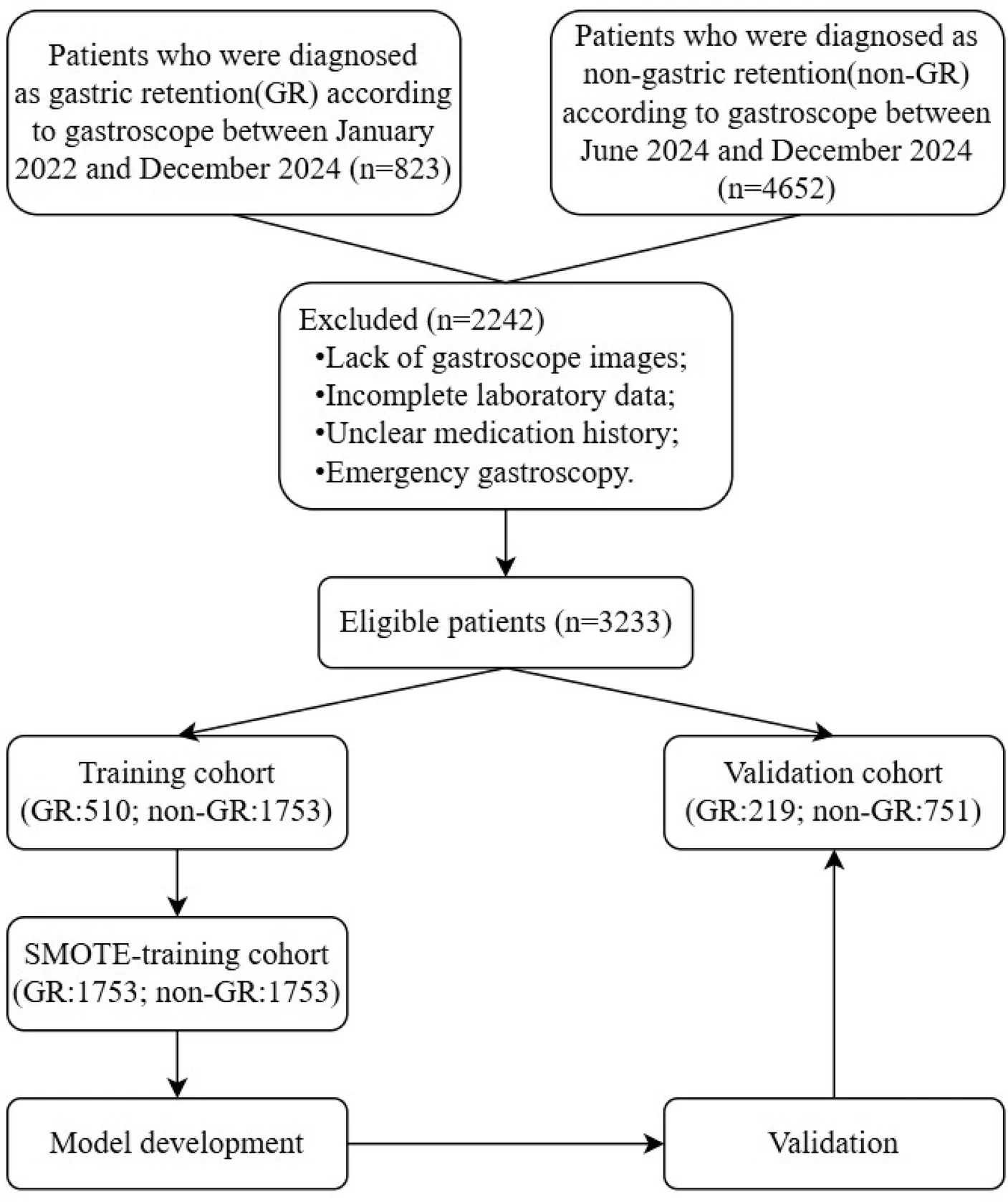
Analytic flowchart of study cohort selection and exclusion criteria.

This retrospective cohort study adhered to the ethical principles outlined in the 1964 Declaration of Helsinki and was approved by the Institutional Review Board of the First Affiliated Hospital of Xiamen University (XMYY-2025KY173). Due to the retrospective nature of the study, written informed consent was abandoned by the ethics committee of the First Affiliated Hospital of Xiamen University. The study adheres to STROBE guidelines. GR was defined as having at least a 6.3% residual volume at four hours or the observation of unexpected food residue after an 8 h fasting protocol during gastroscopy (1, 16). We searched the database of the First Affiliated Hospital of Xiamen University and identified patients diagnosed with GR via gastroscopy between January 2022 and December 2024. Non-GR patients who underwent gastroscopy between June 2024 and December 2024 were also included. All patients undergoing gastroscopy were prepared according to the routine fasting protocol. Patients were excluded based on the following criteria: (1) absence of gastroscopy images, (2) incomplete laboratory data, (3) unclear medication history, and (4) emergency gastroscopy. Consequently, a total of 3,233 patients were ultimately enrolled, consisting of 729 GR patients and 2,504 non-GR patients. Initial clinical information for each patient was extracted from medical records that were documented prior to the index gastroscopy, including basic demographic data (age, gender, education level), surgical history, laboratory data, and medication history. Importantly, predictor variables such as pyloric obstruction, intestinal obstruction, and systemic diseases were ascertained from pre-existing imaging reports, clinical diagnoses, or prior endoscopic records, rather than from the findings of the current index gastroscopy. The study population was randomly divided into a training cohort (*n* = 2,263, with 1,753 non-GR cases and 510 GR cases) and a validation cohort (*n* = 970, with 751 non-GR cases and 219 GR cases) at a ratio of 7:3 via random sampling. The analytic flowchart of the study cohort is illustrated in Figure 1.

### Oversampling

To mitigate class imbalance in the training cohort, we applied the Synthetic Minority Over-sampling Technique (SMOTE) with hyperparameters set to sampling_strategy = “auto”, k_neighbors = 5, and random_state = 42. This method synthesizes new instances that closely emulate the underlying distribution of the minority class. Thereby, a balanced dataset was established which termed the SMOTE-enhanced training cohort, comprising both authentic and algorithmically generated samples (17). The validation cohort was not subjected to SMOTE and remained in its original imbalanced distribution for unbiased final evaluation.

### Feature selection, model development, and validation

To identify clinically relevant predictors within the SMOTE-enhanced training cohort (*n* = 3,506), we performed least absolute shrinkage and selection operator (LASSO) regression after SMOTE oversampling, with the penalty parameter optimized via 10-fold cross-validation. Variance inflation factor (VIF) and pairwise Pearson correlation matrix were calculated for LASSO-selected predictors to evaluate multicollinearity; a VIF threshold of 5 was adopted to define potential multicollinearity. The selected features were subsequently used to develop predictive models using nine distinct machine learning approaches: logistic regression (LR), decision tree (DT), random forest (RF), support vector machine (SVM), extreme gradient boosting (XGBoost), light gradient boosting machine (LightGBM), K-nearest neighbors (KNN), naive Bayes (NB), and artificial neural network (ANN). Hyperparameters for each algorithm were determined via grid search with 10-fold cross-validation on the SMOTE-enhanced training cohort. The final hyperparameter settings are listed in Supplementary Table S1. All models were evaluated in the validation cohort.

The predictive performance of each model was thoroughly assessed using metrics relevant to clinical discrimination and calibration, including the area under the receiver operating characteristic curve (AUC), accuracy, sensitivity, specificity, positive predictive value (PPV), negative predictive value (NPV), confusion matrix analysis, calibration curves, and decision curve analysis (DCA). The optimal probability threshold for the best-performing model was determined using the Youden index. Statistical comparisons of AUC values were conducted using the DeLong test. To ensure robustness, the optimal model underwent further internal validation via 10-fold cross-validation within the SMOTE-enhanced training cohort. Finally, feature importance was interpreted and model outputs were visualized using SHapley Additive exPlanations (SHAP) to enhance clinical interpretability.

### Statistical analysis

Statistical calculations were performed using multiple software packages, including IBM SPSS Statistics (Version 22.0; https://www.ibm.com/spss), R (Version 4.4.1; https://www.r-project.org), and Python (Version 3.7.0; https://www.python.org/). All qualitative variables were analyzed using the chi-square test. The threshold for statistical significance was set at *P* < 0.05 (two-tailed). Within the R environment, all necessary packages can be downloaded from https://cran.r-project.org/web/packages/.

## Results

### Baseline characteristics

Table 1 summarizes the baseline information of all patients in the study. The training cohort consisted of 2,263 patients, including 510 GR cases, whereas the validation cohort comprised 970 patients, with 219 GR cases. No statistically significant differences (*P* > 0.05) were observed between the training and validation cohorts in any characteristics. As shown in Table 2, within the training cohort, significant differences were noted between the non-GR and GR groups regarding age, gender, pyloric obstruction, dyspepsia, history of gastrointestinal surgery, systemic diseases, diabetes, thyroid disorders, hypoalbuminemia, hyponatremia, use of anticholinergic drugs, and use of hypoglycemic agents. In the validation cohort, significant differences were observed in age, BMI, pyloric obstruction, systemic diseases, diabetes mellitus, thyroid disorders, hypoproteinemia, hyponatremia, use of anticholinergic agents, and use of hypoglycemic agents. No statistically significant differences were observed in other characteristics between the two groups (*P* > 0.05).

**Table 1:** Baseline characteristics of patients.

Characteristics	Total (*n* = 3,233)	Training cohort (*n* = 2,263)	Validation cohort (*n* = 970)	*P* value
Group (non-GR/GR)	2,504/729	1,753/510	751/219	0.980
Gender (male/female)	1,263/1,970	895/1,368	368/602	0.390
Age (<65 years/≥65 years)	2,305/928	1,604/659	701/269	0.424
BMI (normal/<18.5 or >28)	2,520/713	1,759/504	761/209	0.649
Educational attainment (junior/senior)	1,434/1,799	1,000/1,263	434/536	0.772
Pyloric obstruction (absence/prsence)	2,899/334	2026/237	873/97	0.686
Intestinal obstruction (absence/prsence)	2,993/240	2,094/169	899/71	0.883
Dyspepsia (absence/prsence)	2,903/330	2,038/225	865/105	0.448
History of gastrointestinal surgery (absence/prsence)	2,701/532	1,905/358	796/174	0.372
Systemic disease (absence/prsence)	2,039/1,194	1,420/843	619/351	0.565
Diabetes mellitus (absence/prsence)	2,676/557	1,881/382	795/175	0.423
Thyroid disorders (absence/prsence)	2,978/255	2,084/179	894/76	0.942
Hypoproteinemia (absence/prsence)	2,880/353	2018/245	862/108	0.797
Hypokalemia (absence/prsence)	2,891/342	2,039/224	852/118	0.055
Hyponatremia (absence/prsence)	2,932/301	2,060/203	872/98	0.310
Use of anticholinergic agents (absence/prsence)	2,371/862	1,712/551	659/311	0.356
Use of analgesics (absence/prsence)	2,378/855	1,660/603	718/252	0.694
Use of hypoglycemic agents (absence/prsence)	3,016/217	2,117/146	899/71	0.366
Use of antihistamines (absence/prsence)	2,968/265	2,085/178	883/87	0.295
Use of sedatives (absence/prsence)	2,597/636	1,809/454	788/182	0.395

GR, gastric retention.

**Table 2:** Comparison of clinical characteristics between two non-GR and GR groups in the training and validation cohorts.

Characteristics	Training cohort			Validation cohort		
	Non-GR (*n* = 1,753)	GR (*n* = 510)	*P* value	Non-GR (*n* = 751)	GR (*n* = 219)	*P* value
Gender (male/female)	716/1,037	179/331	0.019^a^	286/465	82/137	0.864
Age (<65 years/≥65 years)	1,266/487	338/172	0.009^a^	557/194	144/75	0.014^a^
BMI (normal/<18.5 or >28)	1,374/379	385/125	0.167	604/147	157/62	0.006^a^
Educational attainment (junior/senior)	775/978	225/285	0.971	335/416	99/120	0.875
Pyloric obstruction (absence/prsence)	1,624/129	402/108	<0.001^a^	697/54	176/43	<0.001^a^
Intestinal obstruction (absence/prsence)	1,619/134	475/35	0.555	696/55	203/16	0.993
Dyspepsia (absence/prsence)	1,567/186	471/39	0.049^a^	665/86	200/19	0.245
History of gastrointestinal surgery (absence/prsence)	1,478/275	405/106	0.046^a^	638/113	181/38	0.054
Systemic disease (absence/prsence)	1,168/585	252/258	<0.001^a^	511/240	108/111	<0.001^a^
Diabetes mellitus (absence/prsence)	1,500/253	381/129	<0.001^a^	637/114	158/61	<0.001^a^
Thyroid disorders (absence/prsence)	1,633/120	451/59	<0.001^a^	700/51	194/25	0.025^a^
Hypoproteinemia (absence/prsence)	1,602/151	416/94	<0.001^a^	678/73	184/35	0.010^a^
Hypokalemia (absence/prsence)	1,586/167	453/57	0.272	663/88	189/30	0.430
Hyponatremia (absence/prsence)	1,615/138	445/65	<0.001^a^	693/58	179/40	<0.001^a^
Use of anticholinergic agents (absence/prsence)	1,295/458	354/156	0.011^a^	568/183	144/75	<0.001^a^
Use of analgesics (absence/prsence)	1,298/455	362/148	0.168	555/196	163/56	0.875
Use of hypoglycemic agents (absence/prsence)	1,680/73	437/73	<0.001^a^	714/37	185/34	<0.001^a^
Use of antihistamines (absence/prsence)	1,624/129	461/49	0.097	691/60	192/27	0.048^a^
Use of sedatives (absence/prsence)	1,397/356	412/98	0.588	605/146	183/36	0.317

GR, gastric retention.

a Statistically significant at *P* < 0.05.

### Clinical characteristics in the SMOTE-enhanced training cohort

Table 3 shows the clinical characteristics of patients in the SMOTE-enhanced training cohort. Notable differences were identified between the two groups regarding BMI, pyloric obstruction, intestinal obstruction, dyspepsia, history of gastrointestinal surgery, systemic disease, diabetes mellitus, hypoproteinemia, hypokalemia, use of anticholinergic agents, use of hypoglycemic agents, and use of antihistamines (*P* < 0.05). No statistically significant differences were found in other characteristics (*P* > 0.05).

**Table 3:** Comparison of patients’ clinical characteristics between non-GR and GR in the SMOTE-enhanced training cohort.

Characteristics	Non-GR (*n* = 1,753)	GR (*n* = 1,753)	*P* value
Gender (male/female)	716/1,037	678/1,075	0.190
Age (<65 years/≥65 years)	1,266/487	1,251/502	0.573
BMI (normal/<18.5 or >28)	1,374/379	1,446/307	0.002^a^
Educational attainment (junior/senior)	775/978	818/935	0.145
Pyloric obstruction (absence/prsence)	1,624/129	1,374/379	<0.001^a^
Intestinal obstruction (absence/prsence)	1,619/134	1,696/57	<0.001^a^
Dyspepsia (absence/prsence)	1,567/186	1,676/77	<0.001^a^
History of gastrointestinal surgery (absence/prsence)	1,478/275	1,332/421	<0.001^a^
Systemic disease (absence/prsence)	1,168/585	953/800	<0.001^a^
Diabetes mellitus (absence/prsence)	1,500/253	1,318/435	<0.001^a^
Thyroid disorders (absence/prsence)	1,633/120	1,640/113	0.635
Hypoproteinemia (absence/prsence)	1,602/151	1,532/221	<0.001^a^
Hypokalemia (absence/prsence)	1,586/167	1,635/118	0.002^a^
Hyponatremia (absence/prsence)	1,615/138	1,603/150	0.460
Use of anticholinergic agents (absence/prsence)	1,295/458	1,116/637	<0.001^a^
Use of analgesics (absence/prsence)	1,298/455	1,311/442	0.615
Use of hypoglycemic agents (absence/prsence)	1,680/73	1,552/201	<0.001^a^
Use of antihistamines (absence/prsence)	1,624/129	1,661/92	0.010^a^
Use of sedatives (absence/prsence)	1,397/356	1,439/314	0.071

GR, gastric retention; SMOTE, synthetic minority over-sampling technique.

a Statistically significant at *P* < 0.05.

### Feature selection and model development

**Figure 2:**
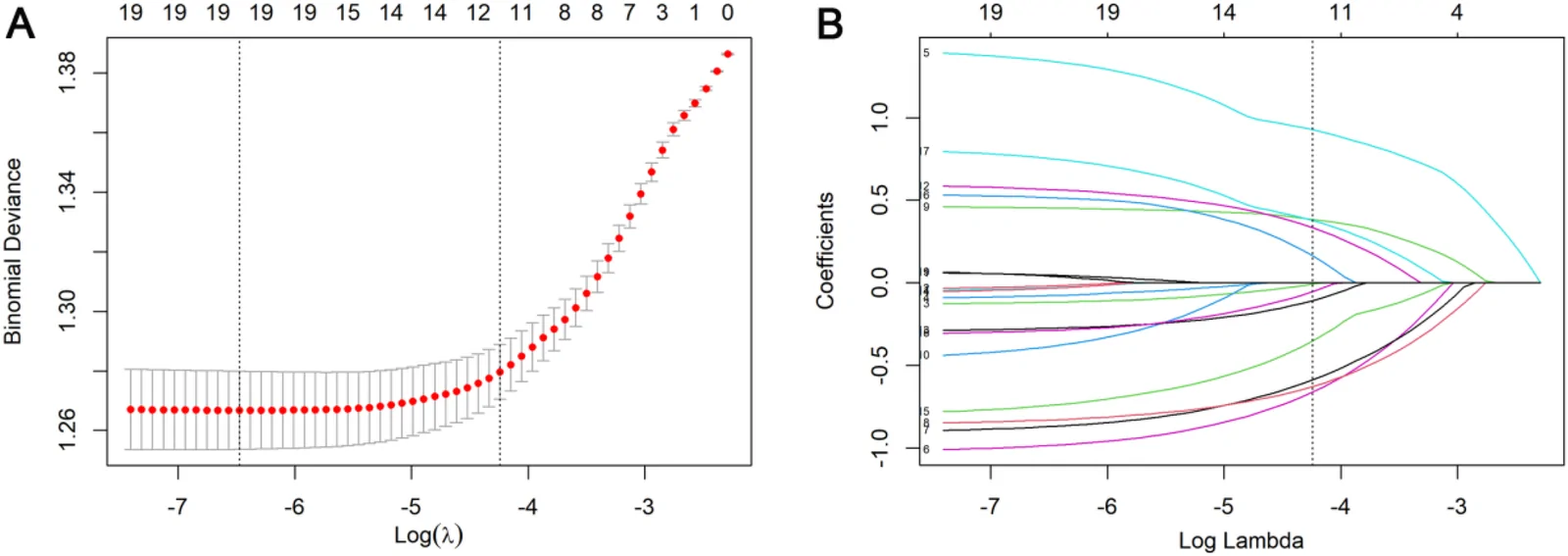
Feature selection using LASSO regression. **(A)** The LASSO tuning parameter (*λ*) was optimized via 10-fold cross-validation combined with the 1 standard error rule. Dotted vertical lines mark the optimal *λ* values, with *λ* = 0.014 (log(*λ*) = −4.243) chosen as the best fit, as shown on the right. **(B)** LASSO coefficient profiles for 19 clinical features were plotted against the selected log(*λ*) from 10-fold cross-validation. This analysis identified 12 features with significant non-zero coefficients, which were then selected for the final model. LASSO, least absolute shrinkage and selection operator.

Upon conducting LASSO regression analysis on the SMOTE-enhanced training cohort, the minimum-*λ* rule retained 19 features, whereas the 1-standard-error rule retained 12 features. Considering model parsimony and clinical interpretability, we selected the 1-standard-error rule with *λ* = 0.014 and log(*λ*) = −4.243 (Figure 2). The 12 features retained under this criterion were: use of anticholinergic agents, pyloric obstruction, use of analgesics, systemic disease, history of gastrointestinal surgery, dyspepsia, intestinal obstruction, hypoproteinemia, BMI, use of antihistamines, diabetes mellitus, and hypokalemia. Multicollinearity diagnostics for the 12 predictors showed that all VIF values ranged from 1.043 to 2.294 and were below 5 (Supplementary Table S2). Pairwise correlation analysis demonstrated weak correlations between variables, and no substantial multicollinearity was observed (Supplementary Figure S1). Subsequently, multiple machine learning-based prediction models were constructed using these 12 selected features.

### Performance of different models

**Figure 3:**
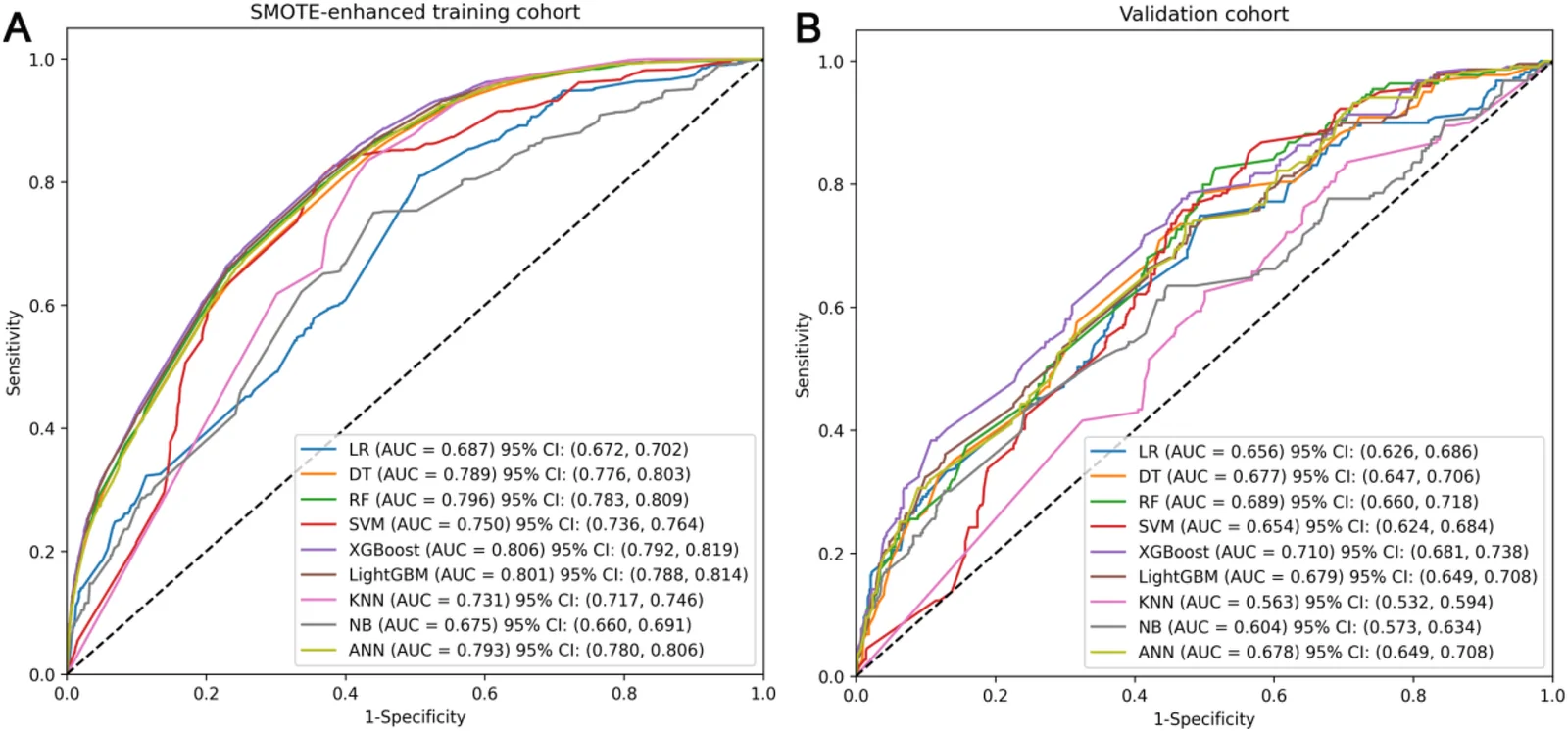
ROC curves of the different ML models. **(A)** SMOTE-enhanced training cohort. **(B)** Validation cohort. ML, machine learning; ROC, receiver operating characteristic curve; SMOTE, synthetic minority oversampling technique; AUC, area under curve; LR, logistic regression; DT, decision tree; RF, random forest; SVM, support vector machine; XGBoost, extreme gradient boosting; LightGBM, light gradient boosting machine; KNN, K-nearest neighbors; NB, naive Bayes; ANN, artificial neural network.

**Figure 4:**
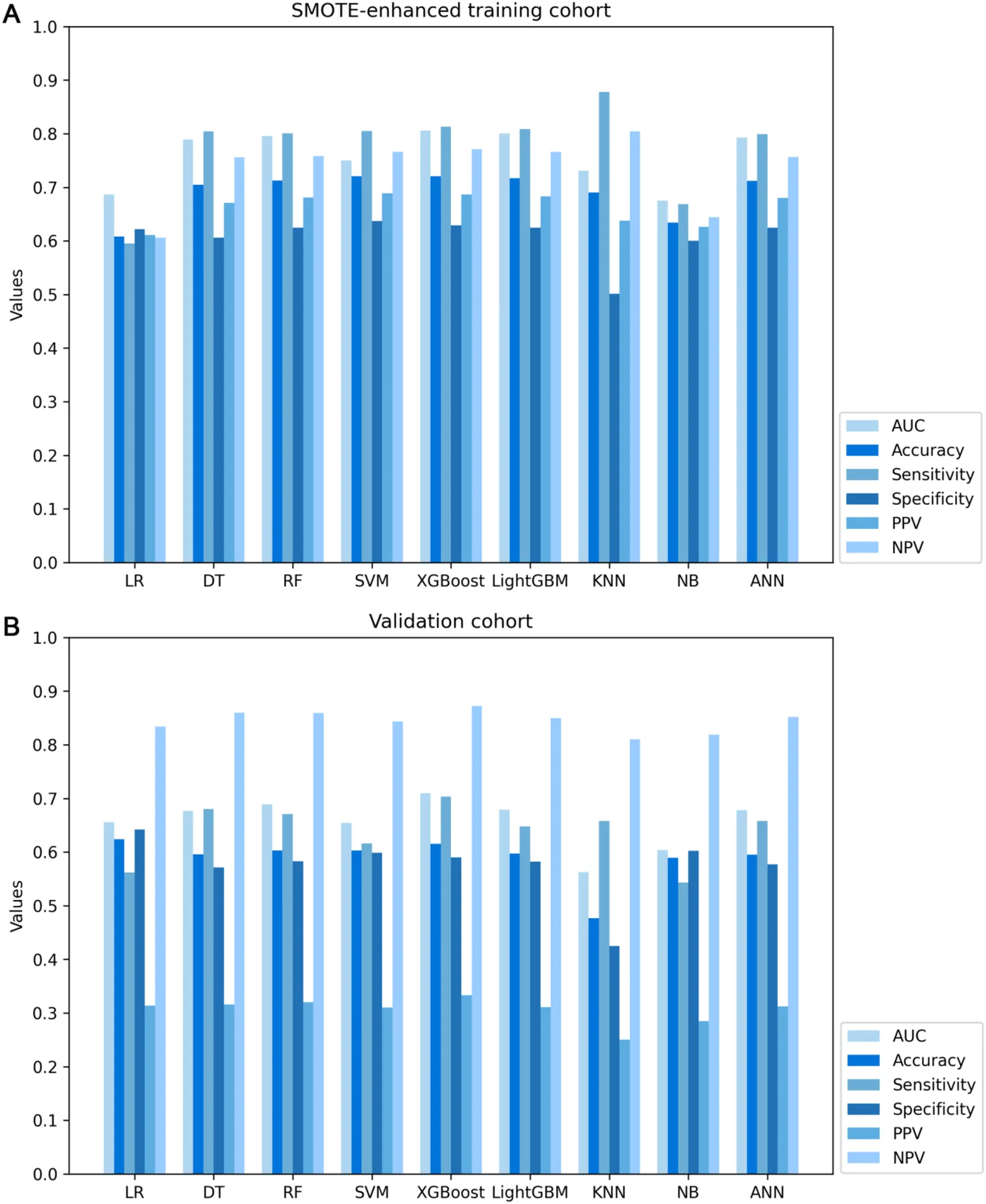
Performances of the different ML models. **(A)** SMOTE-enhanced training cohort. **(B)** Validation cohort. ML, machine learning; SMOTE, synthetic minority oversampling technique; AUC, area under receiver operating characteristics; CI, confidence interval; Acc, accuracy; Sen, sensitivity; Spe, specificity; PPV, positive predictive value; NPV, negative predictive value; LR, logistic regression; DT, decision tree; RF, random forest; SVM, support vector machine; XGBoost, extreme gradient boosting; LightGBM, light gradient boosting machine; KNN, K-nearest neighbors; NB, naive Bayes; ANN, artificial neural network. All metrics were calculated at a 0.5 threshold for model comparison.

**Figure 5:**
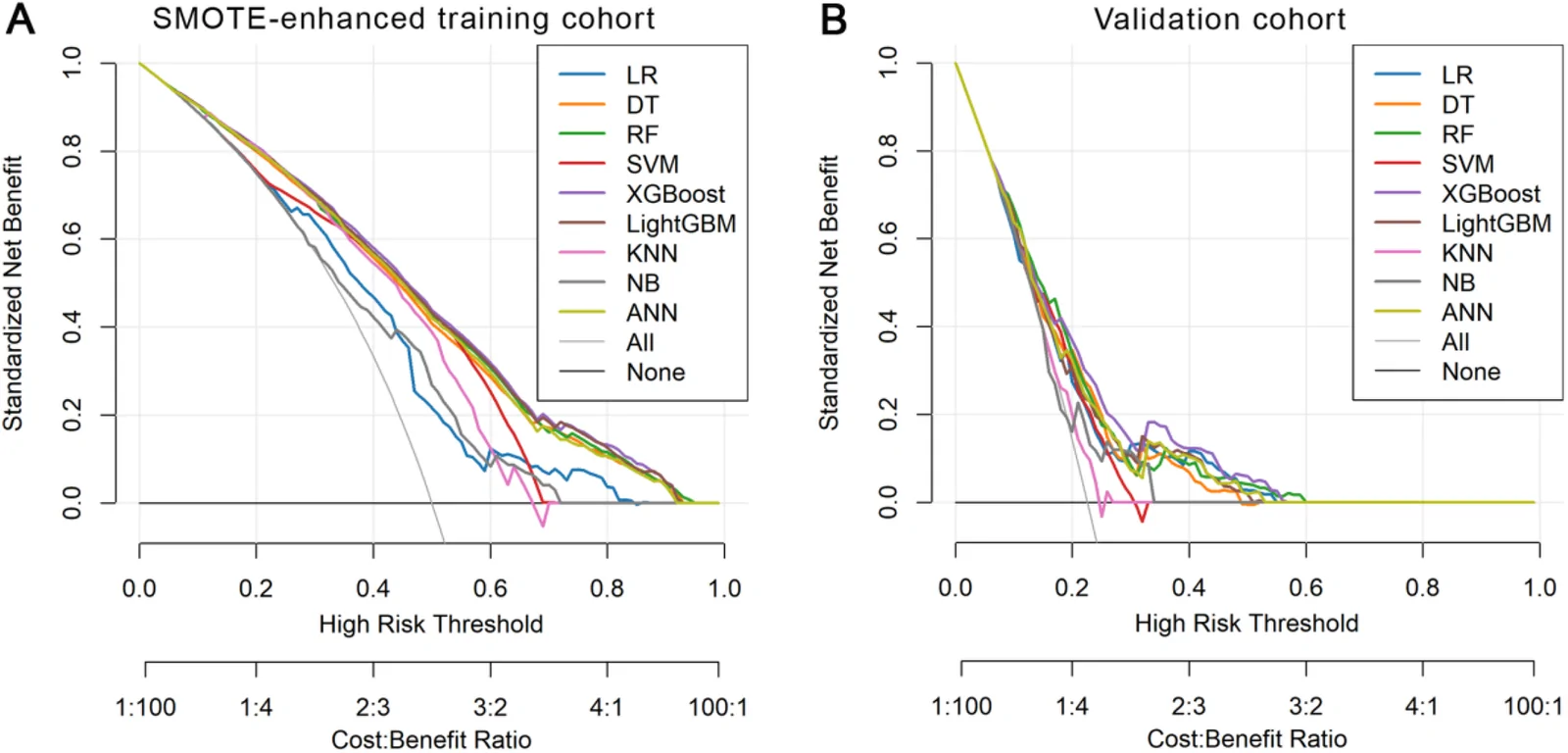
DCA of the different ML models. **(A)** SMOTE-enhanced training cohort. **(B)** Validation cohort. ML, machine learning; DCA, decision curve analysis; SMOTE, synthetic minority oversampling technique; LR, logistic regression; DT, decision tree; RF, random forest; SVM, support vector machine; XGBoost, extreme gradient boosting; LightGBM, light gradient boosting machine; KNN, K-nearest neighbors; NB, naive Bayes; ANN, artificial neural network.

The ROC curve illustrates the performance of each model in the SMOTE-enhanced training cohort and validation cohort, as depicted in Figure 3. Figure 4 and Supplementary Table S3 detail the performance of various prediction models in the SMOTE-enhanced training and validation cohorts, whereas Supplementary Figures S2, S3 depict the corresponding confusion matrices of these models in the SMOTE-enhanced training and validation cohorts. The results indicate that the nine machine learning models achieved AUC ranges of 0.687–0.806 and 0.563–0.710 in the SMOTE-enhanced training cohort and validation cohort, respectively. The XGBoost model demonstrated the best performance in both cohorts. Comparisons of AUC among different prediction models are presented in Supplementary Figure S4. The XGBoost model achieved an average AUC of 0.766 ± 0.019 and an average accuracy of 0.691 ± 0.019 (Supplementary Figure S5) when 10-fold cross-validation was employed in the SMOTE-enhanced training cohort. Calibration curves demonstrated good agreement between predicted and observed probabilities in both cohorts (Supplementary Figure S6), and DCA indicated that this model provided a superior net benefit over the others across a range of threshold probabilities (Figure 5). Threshold-specific performance analysis further revealed that as the probability cutoff increased, sensitivity progressively decreased while specificity improved (Supplementary Figure S7). Based on the Youden index, the optimal probability threshold was determined to be 0.418, yielding a sensitivity of 77.6%, specificity of 53.3%, PPV of 32.6%, and NPV of 89.1% in the validation cohort. Notably, this Youden-optimized cutoff leads to over 45% of GR-negative patients being misclassified as high-risk individuals, which may cause unnecessary prolonged fasting and endoscopy delays. We further calculated three representative alternative cutoffs for differentiated clinical use: a high-sensitivity threshold of 0.278 (sensitivity = 85.4%, specificity = 37.0%), a balanced threshold of 0.510 (sensitivity = 60.3%, specificity = 69.0%), and a high-specificity threshold of 0.624 (sensitivity = 50.7%, specificity = 76.0%). Full numerical results for all candidate thresholds are summarized in Supplementary Table S4.

### Interpretation of the XGBoost model

**Figure 6:**
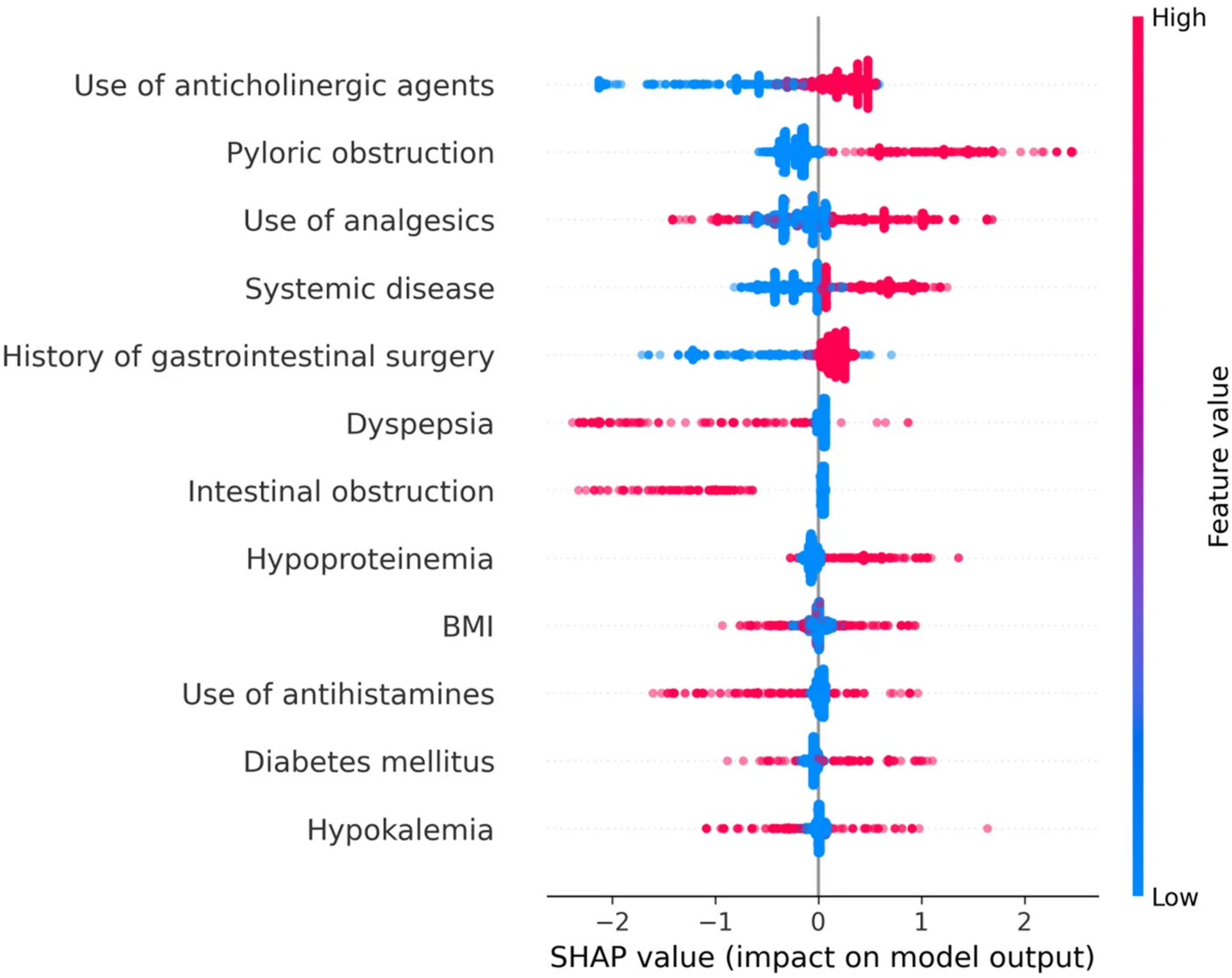
Beeswarm plots derived from the SHAP analysis of the XGBoost model. The plots provide a detailed visualization of how individual feature values contribute to the prediction outcome for each data point within the dataset. Each bee (data point) is color-coded based on the feature value, allowing for a clear identification of the relationship between feature magnitude and its impact on the model's prediction. SHAP, SHapley additive exPlanations; XGBoost, extreme gradient boosting.

**Figure 7:**
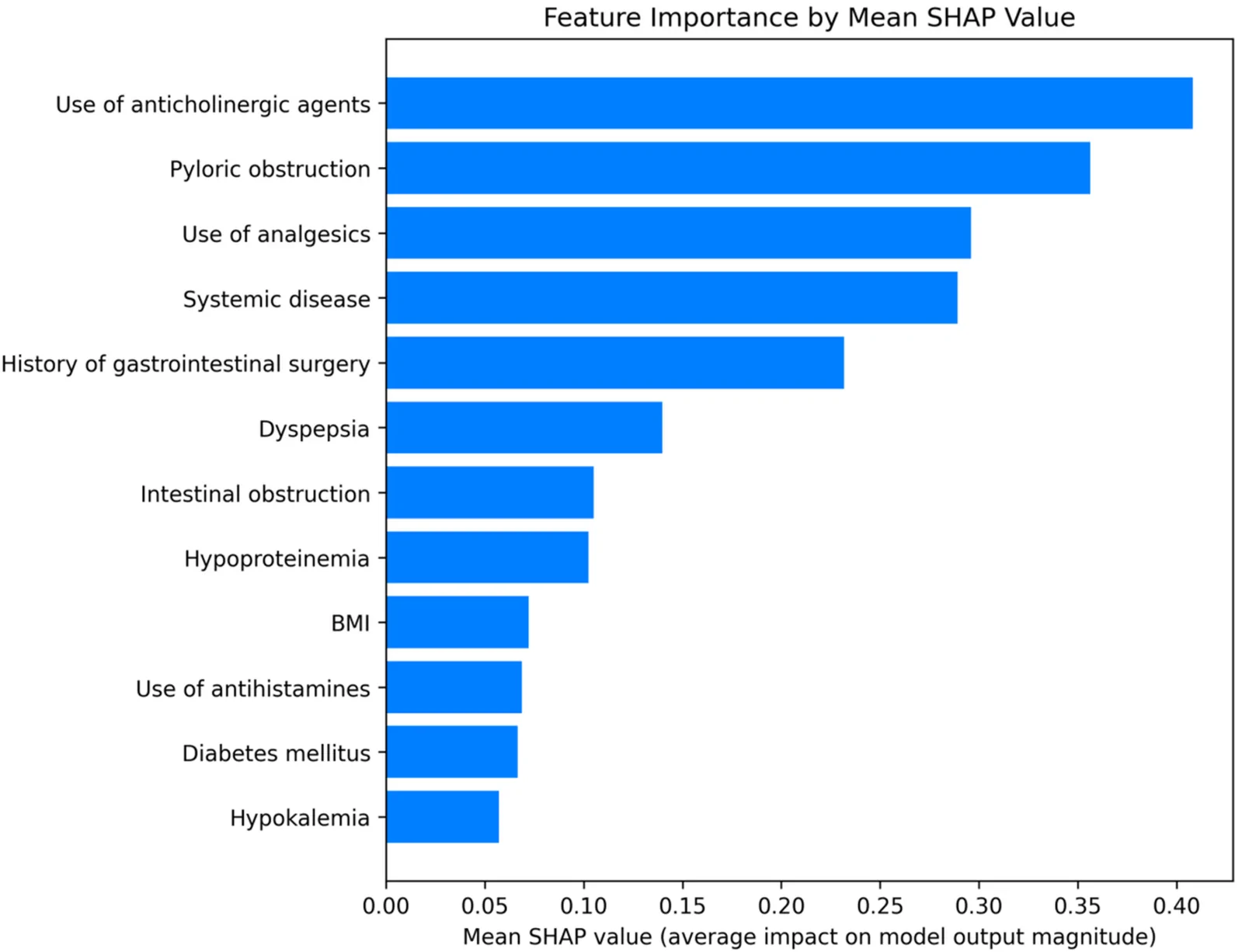
Bar plots from the SHAP analysis of the XGBoost model. The length of each bar represents the overall contribution of a specific feature to the model's predictions across the entire dataset. SHAP, SHapley additive exPlanations; XGBoost, extreme gradient boosting.

**Figure 8:**
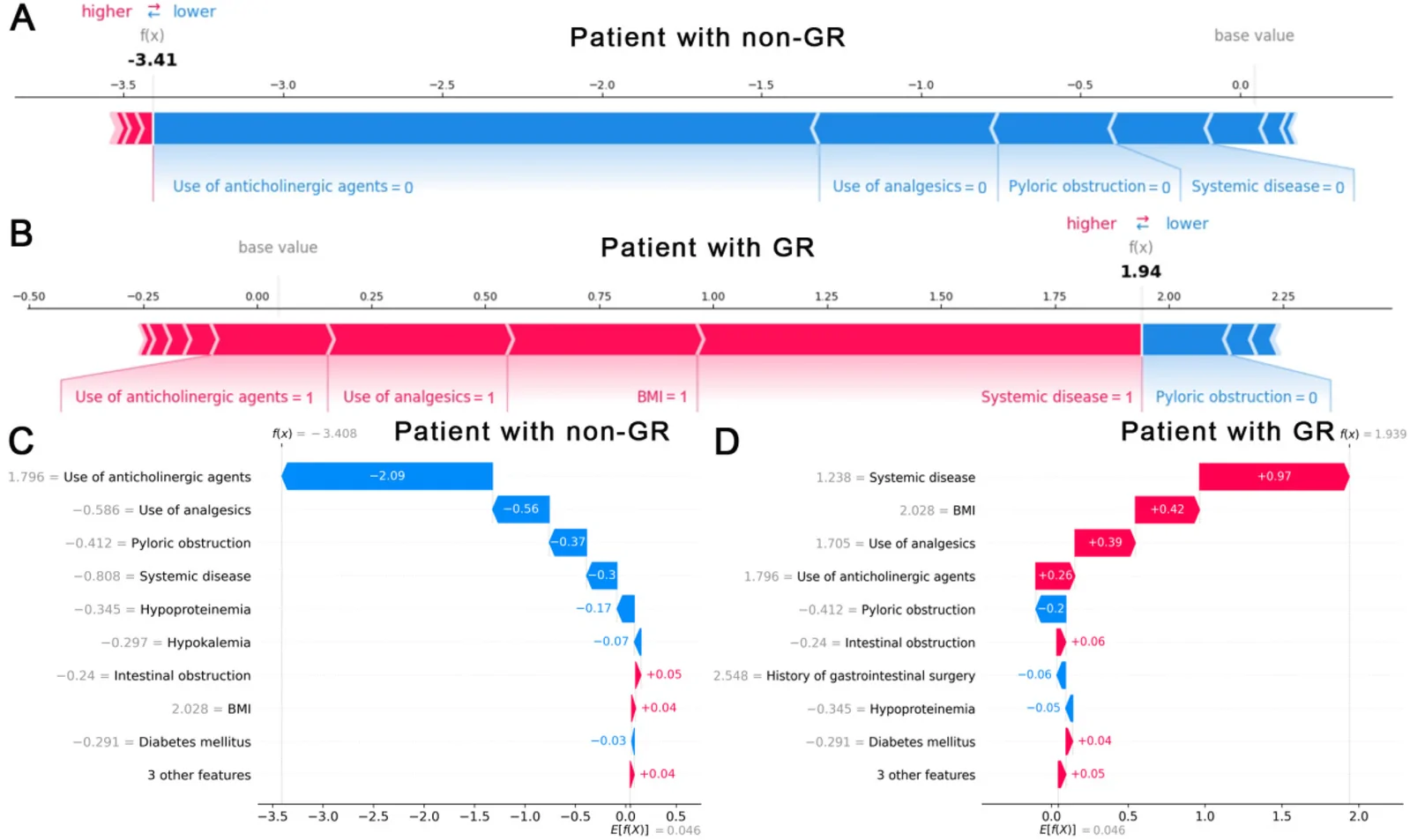
Force plots and waterfall plots depicting the average probabilities for two patients, one with non-GR and one with GR, in the validation cohort. **(A,B)** Force plots offer a patient specific breakdown of how individual features contribute to the predicted probability of the outcome. Each feature is represented by an arrow, where the direction and length of the arrow indicate whether the feature increases or decreases the probability of the outcome and by how much, respectively. **(C,D)** Waterfall plots further expand on the feature impact analysis, showing the sequential contribution of each feature to the probability score. Starting from a baseline probability, each feature is added or subtracted, and the resulting change in probability is visualized as a step in the waterfall. Both types of plots use color-coding to distinguish between positive (red) and negative (blue) contributions of features, enhancing the interpretability of the model's predictions at an individual patient level. GR, gastric retention.

Visualization analysis of the XGBoost model was conducted using the SHAP algorithm. The bar chart indicates that the most influential feature on the model is the use of anticholinergic agents, followed by pyloric obstruction, use of analgesics, systemic disease, and history of gastrointestinal surgery (Figure 6). The beeswarm plot demonstrates that the use of anticholinergic agents, pyloric obstruction, use of analgesics, systemic disease, and history of gastrointestinal surgery are associated with increased GR risk (Figure 7). Figure 8 displays the force plots and waterfall plots for two representative patients (non-GR and GR) from the validation set.

## Discussion

This study represents the large-scale dataset-based study to develop and validate an interpretable ML model for predicting GR risk before gastroscopy. Our analysis of 3,233 patients identified 12 key clinical predictors and demonstrated that the XGBoost algorithm outperformed other ML models, achieving an AUC of 0.806 in the SMOTE-enhanced training cohort and 0.710 in the validation cohort.

The performance of the XGBoost model is consistent with its established strengths in handling complex, non-linear interactions within imbalanced medical datasets. Its AUC of 0.710 in the validation cohort reflects an expected decrease from training performance and falls within the moderate discrimination range. The model's calibration curve showed good agreement between predicted probabilities and observed outcomes, and the DCA curve confirmed its net clinical benefit across a range of threshold probabilities, supporting its potential utility as an auxiliary screening tool, though it should not replace comprehensive clinical judgment. At the optimal probability cutoff of 0.418 (determined by maximizing the Youden index), the model achieved a sensitivity of 77.6% and an NPV of 89.1%, indicating that it could safely rule out GR in nearly 90% of low-risk patients, while the moderate PPV of 32.6% reflects the expected impact of the low disease prevalence (22.6%) on positive predictions. In the validation cohort, XGBoost achieved an AUC of 0.710, compared with 0.656 for logistic regression. XGBoost also showed higher sensitivity and NPV, whereas its accuracy and specificity were not higher than those of logistic regression. Additionally, clinical interpretability via SHAP analysis provides a potentially useful adjunct to traditional risk stratification approaches (12).

We further elaborate on the trade-off between sensitivity and specificity when selecting model thresholds in clinical practice. The threshold of 0.418 derived from the Youden index prioritizes overall statistical performance yet suffers from low specificity (53.3%), producing a high false-positive rate. The balanced cutoff of 0.510 is more suitable for routine gastroscopy scenarios, as it markedly reduces false positive cases while retaining acceptable predictive ability, with balanced sensitivity (60.3%) and specificity (69.0%). For sedated endoscopy where preventing aspiration is the top priority, clinicians can adopt the low threshold of 0.278 to maximize sensitivity (85.4%) and avoid missed GR cases, even though this will greatly increase the number of patients requiring extended fasting. Conversely, the high threshold of 0.624 can be applied in unsedated outpatient endoscopy to lift specificity to 76.0%, easing hospital scheduling burdens at the cost of partially reduced sensitivity. Clinicians can flexibly adjust the probability cutoff according to anesthesia mode, patient conditions and institutional operational demands to balance clinical safety and work efficiency.

We found that the use of anticholinergic agents emerged as the strongest predictor of GR. This is mechanistically plausible, as anticholinergics directly inhibit gastric smooth muscle contraction and delay gastric emptying. Similarly, the use of analgesics, antihistamines, and hypoglycemic agents were also significant contributors. These findings emphasize the crucial role of medication review in GR risk assessment. Pyloric obstruction and a history of gastrointestinal surgery were among the top predictors. Obstruction physically impedes emptying, while surgery can disrupt neural (vagal) or anatomical pathways essential for normal motility (18, 19). A history of gastrointestinal surgery may cause GR by altering normal anatomy and damaging the vagus nerve, leading to abnormal peristalsis and food residue (2, 20–23). However, intestinal obstruction has a lesser impact on GR, possibly because the condition is more severe, and gastroscopy is not a necessary examination for such patients.

The presence of systemic diseases, diabetes mellitus, hypoproteinemia, and hypokalemia were significant predictors. Hypoproteinemia may reflect malnutrition or systemic inflammation affecting motility, while hypokalemia can directly impair smooth muscle function (24). Current evidence regarding BMI and gastric motility remains conflicting. In our study, BMI <18.5 or BMI>28 showed no significant association with gastric retention risk when compared to normal BMI controls. While some studies suggest that low BMI may contribute to gastric retention, others indicate that elevated BMI accelerates gastric emptying rates (25–28). Results from many studies in patients with diabetes indicate that diabetes is a risk factor for gastroparesis (29, 30). However, in our study, diabetes was not among the top five factors affecting GR, possibly because the diabetic patients received timely medication intervention and were advised to extend fasting time.

Several previous studies have evaluated gastric food retention, residual gastric content, or gastroparesis prediction in specific clinical contexts. Coleski et al. and Phillips et al. identified important clinical factors associated with endoscopic gastric food retention or high-risk residual gastric content, but these studies mainly focused on risk-factor identification rather than developing an interpretable machine-learning risk stratification tool for routine pre-gastroscopy use (1, 31). Jia et al. developed a logistic regression-based model for preoperative gastric retention before ERCP, with good discrimination in a small validation cohort; however, ERCP represents a more selected therapeutic endoscopy population, and the sample size was substantially smaller than that of the present study (32). Other prediction models have focused on postsurgical gastroparesis syndrome after gastrectomy or complete mesocolic excision, or on gastroparesis in chronic pancreatitis. Although some of these disease- or surgery-specific models reported higher AUC values, their target populations, endpoints, and predictors differ substantially from those required for routine gastroscopy screening.

Compared with these prior studies, the present model has several practical advantages for the specific task of predicting gastric retention before gastroscopy. First, it was developed in a larger routine gastroscopy cohort and used routinely available pre-procedural variables, making it more suitable for integration into standard endoscopy workflows. Second, using the same LASSO-selected predictors, we evaluated nine algorithms (with LR as baseline). In the validation cohort, XGBoost showed higher discrimination than LR (AUC: 0.710 vs. 0.656), along with superior sensitivity and NPV. Third, the model was evaluated not only by ROC analysis but also by calibration, decision curve analysis, confusion matrices, and SHAP-based interpretation. Therefore, although external validation is still required, the proposed XGBoost model offers a useful reference that is more comprehensive and clinically interpretable for pre-gastroscopy GR risk stratification than prior risk-factor studies or models developed for other disease- or procedure-specific populations.

The integration of machine learning into clinical practice continues to expand, offering robust tools for developing predictive models. These models enable precision medicine through individualized risk stratification and treatment optimization, thereby enhancing patient outcomes (7, 13, 15). By integrating readily available clinical data (demographics, history, medications, basic labs), this model may enable early identification of high-risk patients for GR. High-risk patients could undergo extended fasting protocols, receive prokinetic agents, or have their gastroscopy scheduled as the first procedure of the day. Identifying low-risk patients could potentially allow for more streamlined fasting protocols, improving patient comfort and operational efficiency. Ultimately, this stratification aims to significantly reduce the incidence of aspiration pneumonia and asphyxiation. Currently, challenges such as the integration of electronic health records, automated extraction of drug and laboratory variables, cross-institutional model calibration, and physician training require urgent resolution to optimize endoscopic examination scheduling and anesthesia assessment workflows.

This study has several limitations. Firstly, as a single-center retrospective study, it may have selection bias. This study lacks external independent data validation; therefore, prospective, multi-center validation studies are essential to confirm the model's generalizability and real-world clinical impact. Secondly, factors such as glycated hemoglobin level and gastric emptying scintigraphy were not included in the study due to limited conditions. The inclusion of more detailed clinical data is expected to improve the predictive performance of the model. Third, SMOTE oversampling was performed prior to LASSO feature selection. Artificially synthesized minority samples cannot fully mimic the real distribution of patients with gastric retention, and this analytical pipeline may introduce some degree of optimism during feature selection and model training. Although we conducted 10-fold cross-validation within the SMOTE-enhanced training cohort to assess model stability on the balanced dataset, this cross-validation cannot completely eliminate the bias introduced by pre-analysis oversampling. Future investigations on independent naturally imbalanced cohorts are warranted to further verify the robustness of the identified predictors. Lastly, while SMOTE effectively addressed class imbalance for model training, the synthetic samples may not perfectly reflect the true underlying distribution of the minority GR class. Additionally, the moderate AUC of 0.710 indicates that the current model lacks the precision required for standalone clinical decision-making. Nevertheless, this level of discrimination is comparable to that of existing risk stratification tools for similar clinical scenarios, supporting its potential as an auxiliary screening aid, though further refinement through the inclusion of more comprehensive objective indicators is needed before broader clinical implementation.

## Conclusions

We developed an interpretable XGBoost model using 12 routinely collected clinical features to predict GR risk before gastroscopy. The model showed moderate discrimination with good calibration. SHAP analysis identified anticholinergic use, pyloric obstruction, gastrointestinal surgery history, analgesic use, and systemic disease as key contributing features. The model enables early identification of high-risk patients to guide pre-procedural interventions, but should be viewed as an auxiliary screening aid rather than a definitive diagnostic tool. Future prospective external validation is warranted.

## Data Availability

The original contributions presented in the study are included in the article/Supplementary Material, further inquiries can be directed to the corresponding author/s.
