# Supplementary material for: Interpretable and explainable predictive machine learning models for gastric retention before gastroscopy
Source: Front Med (Lausanne). 2026 Aug 27;13:1898316. doi: 10.3389/fmed.2026.1898316 (PMC13560489; doi:10.3389/fmed.2026.1898316)
Supplement: Supplementary file 1 [file Table1.docx]

**Supplementary Table 1** Hyperparameter settings of nine ML algorithms used for constructing prediction model.

|  | **Hyperparameter settings** |
| --- | --- |
| LR | 'C': 0.1, 'penalty': 'l2' |
| DT | 'criterion': 'gini', 'max_depth': None, 'min_samples_leaf': 4, 'min_samples_split': 2 |
| RF | ''bootstrap': True, 'max_depth': 10, 'min_samples_leaf': 2, 'min_samples_split': 2, 'n_estimators': 300 |
| SVM | 'C': 1, 'gamma': 1, 'kernel': 'rbf' |
| XGBoost | 'colsample_bytree': 1.0, 'learning_rate': 0.1, 'max_depth': 7, 'n_estimators': 100, 'subsample': 0.8 |
| LightGBM | 'bagging_fraction': 0.8, 'feature_fraction': 0.9, 'learning_rate': 0.15, 'num_leaves': 45 |
| KNN | 'metric': 'manhattan', 'n_neighbors': 11, 'weights': 'distance' |
| NB | 'alpha': '1.0', 'it_prior': 'True', 'class_prior': 'None' |
| ANN | 'activation': 'relu', 'alpha': 0.0001, 'hidden_layer_sizes': (50,), 'learning_rate': 'constant', 'random_state': 42, 'solver': 'adam' |

Note: ML, machine learning; LR, logistic regression; DT, decision tree; RF, random forest; SVM, support vector machine; XGBoost, extreme gradient boosting; LightGBM, light gradient boosting machine; KNN, k-nearest neighbors; NB, naive Bayes; ANN, artificial neural network.

**Supplementary Table 2** Variance inflation factors of the LASSO-selected predictors.

| **Variable** | **Variance inflation factor** |
| --- | --- |
| Use of analgesics | 2.294 |
| Use of anticholinergic agents | 2.182 |
| Pyloric obstruction | 1.330 |
| Diabetes mellitus | 1.300 |
| Systemic disease | 1.277 |
| BMI | 1.192 |
| History of gastrointestinal surgery | 1.100 |
| Use of antihistamines | 1.076 |
| Hypoproteinemia | 1.070 |
| Hypokalemia | 1.063 |
| Dyspepsia | 1.054 |
| Intestinal obstruction | 1.043 |

Note: LASSO, least absolute shrinkage and selection operator.

**Supplementary Table 3** Predictive performance of different ML models in the SMOTE-enhanced training and validation cohorts (based on a uniform 0.5 probability threshold).

| ***Model*** | ***AUC(95% CI)*** | ***Acc(%)*** | ***Sen (%)*** | ***Spe (%)*** | ***PPV (%)*** | ***NPV (%)*** |
| --- | --- | --- | --- | --- | --- | --- |
| ***SMOTE-enhanced training cohort*** | | | | | | |
| LR | 0.687(0.672-0.702) | 60.8% | 59.5% | 62.2% | 61.1% | 60.6% |
| DT | 0.789(0.776-0.803) | 70.5% | 80.4% | 60.6% | 67.1% | 75.6% |
| RF | 0.796(0.783-0.809) | 71.3% | 80.1% | 62.5% | 68.1% | 75.8% |
| SVM | 0.750(0.736-0.764) | 72.1% | 80.5% | 63.7% | 68.9% | 76.6% |
| XGBoost | 0.806(0.792-0.819) | 72.1% | 81.3% | 62.9% | 68.7% | 77.1% |
| LightGBM | 0.801(0.788-0.814) | 71.7% | 80.9% | 62.5% | 68.3% | 76.6% |
| LDA | 0.731(0.717-0.746) | 69.0% | 87.8% | 50.1% | 63.8% | 80.4% |
| NB | 0.675(0.660-0.691) | 63.4% | 66.9% | 59.9% | 63.0% | 64.4% |
| ANN | 0.793(0.780-0.806) | 71.2% | 79.9% | 62.5% | 68.0% | 75.7% |
| ***Validation cohort*** | | | | | | |
| LR | 0.656(0.626-0.686) | 62.4% | 56.2% | 64.2% | 31.4% | 83.4% |
| DT | 0.677(0.647-0.706) | 59.6% | 68.0% | 57.1% | 31.6% | 86.0% |
| RF | 0.689(0.660-0.718) | 60.3% | 67.1% | 58.3% | 32.0% | 85.9% |
| SVM | 0.654(0.624-0.684) | 60.3% | 61.6% | 59.9% | 31.0% | 84.3% |
| XGBoost | 0.710(0.681-0.738) | 61.5% | 70.3% | 59.0% | 33.3% | 87.2% |
| LightGBM | 0.679(0.649-0.708) | 59.7% | 64.8% | 58.2% | 31.1% | 85.0% |
| KNN | 0.563(0.532-0.594) | 47.7% | 65.8% | 42.5% | 25.0% | 81.0% |
| NB | 0.604(0.573-0.634) | 58.9% | 54.3% | 60.2% | 28.5% | 81.9% |
| ANN | 0.678(0.649-0.708) | 59.5% | 65.8% | 57.7% | 31.2% | 85.2% |

Note: ML, machine learning; AUC, area under receiver operating characteristics; CI, confidence interval; Acc, accuracy; Sen, sensitivity; Spe, specificity; PPV, positive predictive value; NPV, negative predictive value; SMOTE, synthetic minority over-sampling technique; LR, logistic regression; DT, decision tree; RF, random forest; SVM, support vector machine; XGBoost, extreme gradient boosting; LightGBM, light gradient boosting machine; KNN, K-nearest neighbors; NB, naive Bayes; ANN, artificial neural network.

**Supplementary Table 4** Threshold-specific performance of the XGBoost model in the validation cohort.

| ***Threshold*** | ***Acc(%)*** | ***Sen (%)*** | ***Spe (%)*** | ***PPV (%)*** | ***NPV (%)*** |
| --- | --- | --- | --- | --- | --- |
| 0.278 | 47.9% | 85.4% | 37.0% | 28.3% | 89.7% |
| 0.418 | 58.8% | 77.6% | 53.3% | 32.6% | 89.1% |
| 0.510 | 67.0% | 60.3% | 69.0% | 36.2% | 85.6% |
| 0.624 | 70.3% | 50.7% | 76.0% | 38.1% | 84.1% |

Note: XGBoost, extreme gradient boosting; Acc, accuracy; Sen, sensitivity; Spe, specificity; PPV, positive predictive value; NPV, negative predictive value.

**Supplementary Figure 1** Pairwise correlation matrix of predictors selected by LASSO regression. Note: LASSO, least absolute shrinkage and selection operator.

**Supplementary Figure 2** Confusion matrices of different ML models in the SMOTE-enhanced training cohort. **(A)** LR model. **(B)** DT model. **(C)** RF model. **(D)** SVM model. **(E)** XGBoost model. **(F)** LightGBM model. **(G)** KNN model. **(H)** NB model. **(I)** ANN model. Note: ML, machine learning; SMOTE, synthetic minority over-sampling technique; LR, logistic regression; DT, decision tree; RF, random forest; SVM, support vector machine; XGBoost, extreme gradient boosting; LightGBM, light gradient boosting machine; KNN, K-nearest neighbors; NB, naive Bayes; ANN, artificial neural network. Confusion matrices were based on a 0.5 threshold.

**Supplementary Figure 3** Confusion matrices of different ML models in the validation cohort. **(A)** LR model. **(B)** DT model. **(C)** RF model. **(D)** SVM model. **(E)** XGBoost model. **(F)** LightGBM model. **(G)** KNN model. **(H)** NB model. **(I)** ANN model. Note: ML, machine learning; LR, logistic regression; DT, decision tree; RF, random forest; SVM, support vector machine; XGBoost, extreme gradient boosting; LightGBM, light gradient boosting machine; KNN, K-nearest neighbors; NB, naive Bayes; ANN, artificial neural network. Confusion matrices were based on a 0.5 threshold.

**Supplementary Figure 4** Comparison of the AUC values of different ML models in the SMOTE-enhanced training and validation cohorts. **(A)** SMOTE-enhanced training cohort. **(B)** Validation cohort. Note: AUC, area under receiver operating characteristics; ML, machine learning; SMOTE, synthetic minority over-sampling technique; LR, logistic regression; DT, decision tree; RF, random forest; SVM, support vector machine; XGBoost, extreme gradient boosting; LightGBM, light gradient boosting machine; KNN, K-nearest neighbors; NB, naive Bayes; ANN, artificial neural network.

**Supplementary Figure 5** The AUC and accuracy obtained from the 10-fold cross-validation of the XGBoost model in the SMOTE-enhanced training cohort. Note: AUC, area under curve; XGBoost, extreme gradient boosting; SMOTE, synthetic minority over-sampling technique.

**Supplementary Figure 6** Calibration curves of the XGBoost model. Calibration curves for the XGBoost model in SMOTE-enhanced training **(A)** and validation **(B)** cohorts were generated to evaluate model goodness of fit. The 45° line represents perfect prediction. Closer curves imply higher accuracy, indicating better real world predictive performance. Note: XGBoost, extreme gradient boosting; SMOTE, synthetic minority oversampling technique.

**Supplementary Figure 7** Threshold-specific performance of the XGBoost model in the validation cohort. **(A)** Sensitivity and specificity vs. probability threshold, with the optimal cutoff (0.418) marked by the green dashed line. **(B)** Youden index vs. probability threshold, with the maximum value (0.309) indicated by the red dot.
